# Interim Adjusted Estimates of Seasonal Influenza Vaccine Effectiveness — United States, February 2013

**Published:** 2013-02-22

**Authors:** Lisa Jackson, Michael L. Jackson, C. Hallie Phillips, Joyce Benoit, Edward A. Belongia, Deanna Cole, Sarah Kopitzke, Tamara A. Kronenwetter Koepel, Huong Q. McLean, Jennifer K. Meece, Sandra K. Strey, Maria E. Sundaram, Mary Vandermause, Manjusha Gaglani, Juhee Song, Lydia Clipper, Dean Kjar, Anne Robertson, Kempapura Murthy, Melinda Dunnahoo, Stephanie Oliver, Monica Weir, Hope Gonzales, Martha Zayed, Teresa Ponder, JoAnn Nichols, Michael Reis, Cathleen Rivera, David Morgan, Pedro Piedra, Vasanthi Avadhanula, Arnold S. Monto, Suzanne E. Ohmit, Joshua G. Petrie, Emileigh Johnson, Rachel T. Cross, Casey Martens, Marcus Zervos, Lois Lamerato, Mary Ann Aubuchon, Gregory G. Wolff, Heather Eng, Mary Patricia Nowalk, Stephen R. Wisniewski, Richard K. Zimmerman, Charles R. Rinaldo, Arlene Bullotta, Joe Suyama, Evelyn Reis, Donald B. Middleton, Rachel Hess, Jonathan M. Raviotta, Mark G. Thompson, Alicia M. Fry, Swathi N. Thaker, Jill Ferdinands, Po-Yung Cheng, Sarah Spencer, Erin Burns, LaShondra Berman, Wendy Sessions, Angie Foust, Joseph Bresee, Nancy Cox

**Affiliations:** Group Health Research Institute, Seattle, Washington; Marshfield Clinic Research Foundation, Marshfield, Wisconsin; Scott and White Healthcare, Temple, and Baylor College of Medicine, Houston, Texas; Univ of Michigan, Ann Arbor, and Henry Ford Health System, Detroit, Michigan; Univ of Pittsburgh Schools of the Health Sciences and Univ of Pittsburgh Medical Center, Pittsburgh, Pennsylvania; Influenza Div, CDC

Early influenza activity during the 2012–13 season (1) enabled estimation of the unadjusted effectiveness of the seasonal influenza vaccine (2). This report presents updated adjusted estimates based on 2,697 children and adults enrolled in the U.S. Influenza Vaccine Effectiveness (Flu VE) Network during December 3, 2012–January 19, 2013. During this period, overall vaccine effectiveness (VE) (adjusted for age, site, race/ethnicity, self-rated health, and days from illness onset to enrollment) against influenza A and B virus infections associated with medically attended acute respiratory illness was 56%, similar to the earlier interim estimate (62%) (2). VE was estimated as 47% against influenza A (H3N2) virus infections and 67% against B virus infections. When stratified by age group, the point estimates for VE against influenza A (H3N2) and B infections were largely consistent across age groups, with the exception that lower VE against influenza A (H3N2) was observed among adults aged ≥65 years. These adjusted VE estimates indicate that vaccination with the 2012–13 influenza season vaccine reduced the risk for outpatient medical visits resulting from influenza by approximately one half to two thirds for most persons, although VE was lower and not statistically significant among older adults. Antiviral medications should be used as recommended for treatment of suspected influenza in certain patients, including those aged ≥65 years, regardless of their influenza vaccination status.

Details of the VE network design, sites, and enrollment procedures have been described previously (2,3). In this report, patients aged ≥6 months seeking outpatient medical care for an acute respiratory illness with cough, within 7 days of illness onset, were enrolled at five study sites.* Consenting participants completed an enrollment interview. Nasal and oropharyngeal swabs were combined and tested using CDC’s real-time reverse transcription–polymerase chain reaction (rRT-PCR) protocol. Participants were considered vaccinated if they had received ≥1 dose of any seasonal influenza vaccine ≥14 days before illness onset, according to medical records and registries (at Texas, Washington, and Wisconsin sites) or self-report (at Michigan and Pennsylvania sites).

Of the 2,697 children and adults enrolled during December 3, 2012–January 19, 2013, a total of 1,115 (41%) tested positive for influenza virus by rRT-PCR (Figure). The proportion of patients with influenza differed by study site, sex, age group, race/ethnicity, self-rated health status, and interval from illness onset to enrollment (Table 1). The proportion vaccinated ranged from 36% to 54% across sites and also differed by sex, age group, race/ethnicity, and self-rated health status (Table 1).

Among the patients with influenza, 32% had been administered the 2012–13 seasonal influenza vaccine, compared with 50% of the influenza-negative controls (Table 2). For all persons with medically attended acute respiratory illness, the overall VE (adjusted for age group, study site, race/ethnicity, self-rated health status, and days from illness onset to enrollment) against influenza A and B virus infections was 56% (95% confidence interval [CI] = 47%–63%) (Table 2). Significant VE against influenza A and B viruses was observed among persons in all age groups, except for adults aged ≥65 years.

Among the 751 infections with influenza A viruses, 560 (75%) had been subtyped; 546 (98%) of the infections were caused by influenza A (H3N2) viruses (Table 1). The adjusted VE for all ages against influenza A (H3N2) virus infection was 47% (CI = 35%–58%) (Table 2). The adjusted, age-stratified VE point estimates were 58% for persons aged 6 months–17 years, 46% for persons aged 18–49 years, 50% for persons aged 50–64 years, and 9% for persons aged ≥65 years (Table 2).

A total of 366 (33%) of the 1,115 cases had infections caused by influenza B viruses (Table 1). The adjusted VE estimate for all ages against influenza B was 67% (51%–78%) (Table 2). The adjusted VE point estimates against influenza B ranged from 64% to 75% across age groups.

## Editorial Note

These updated and age-adjusted VE estimates for the 2012–13 influenza vaccine confirm moderate effectiveness in preventing outpatient medical visits caused by circulating influenza viruses, similar to earlier unadjusted estimates in the United States (2) and to recent interim estimates from Canada and Europe (4,5). Overall, influenza vaccination reduced the risk for medical visits resulting from influenza A and B by 56%, from influenza A (H3N2) by 47%, and from influenza B by 67%. The preventive benefits against influenza B were consistent across age groups. The adjusted VE estimates against influenza A (H3N2) viruses also were largely consistent (46%–58%) for persons aged 6 months–64 years, but the estimate was not significant among persons aged ≥65 years. These VE estimates are not final; an increased sample size and adjustment for additional potential confounders (such as chronic medical conditions and functional status) at the end of the season could change these estimates.

Confirmation of the protective benefits of the 2012–13 influenza vaccine among persons aged 6 months–64 years offers further support for the public health benefit of annual seasonal influenza vaccination and supports the expansion of vaccination, particularly among younger age groups. The nonsignificant adjusted VE of 9% against A (H3N2) among persons aged ≥65 years is similar to the estimate in a recent interim report from Europe (6) and reinforces the need for continued advances in influenza vaccines, especially to increase protective benefits for older adults.

One possible explanation for these findings is that some older adults did not mount an effective immune response to the influenza A (H3N2) component of this season’s vaccine. Nonetheless, this finding should not discourage future vaccination by persons aged ≥65 years, who are at greater risk for more severe cases and complications from influenza. Influenza vaccines remain the best preventive tool available, and VE is known to vary by virus type/subtype, age group, season, host immunity, and the outcome measured (7). This study observed a VE point estimate against influenza B (67%) that was much higher than the 9% VE estimate against A (H3N2) among older adults, although the precision of estimates was limited by the small sample. Although some previous studies have shown influenza vaccine benefits for older adults, others have failed to demonstrate statistically significant benefits against specific influenza types or subtypes (7). Variability among studies and across seasons and age groups is to be expected and should not change recommendations for annual vaccination. It is also important to note that the VE estimates in this report are limited to the prevention of outpatient medical visits, rather than more severe illness outcomes, such as hospitalization or death. A previous multiseason study found that the influenza vaccine reduced the risk for influenza-associated hospitalizations among older adults by 61% (CI = 18%–82%) (8). A full evaluation of the VE for older adults this season must await consideration of additional data and outcomes.

Clinicians should maintain a high index of suspicion for influenza infection among persons with acute respiratory illness while influenza activity is ongoing. Early antiviral treatment can reduce influenza-associated illness severity and complications (9); this season, antiviral treatment of elderly adults is especially important.† CDC recommends initiating antiviral medications for patients with suspected influenza, regardless of their influenza vaccination status, if they are aged ≥65 years, or hospitalized, or have progressive or complicated illness, or otherwise are at higher risk for complications from influenza.§ Antiviral treatment can be initiated empirically, preferably within 48 hours after illness onset, and should not be delayed pending confirmatory diagnostic testing nor be dependent upon tests with limited sensitivity (e.g., negative rapid tests). Among hospitalized patients, treatment should be initiated on admission; several studies suggest effectiveness of antiviral treatment even when initiated ≥48 hours after illness onset (9).

The findings in this report are subject to at least four limitations. First, the observational study design has greater potential for confounding and bias relative to randomized clinical trials. Second, although these midseason VE estimates were adjusted for potential confounders identified in previous studies (3), additional factors will be considered in final end-of-season estimates, including health-care–seeking behavior, differences in functional status, and severity of illness, which could influence VE estimates, especially for older adults. Third, no adjustment was made for chronic medical conditions, because of a lack of medical record data for interim analyses; however, VE estimates were adjusted for self-rated health, which is associated with chronic illness and mortality risk (10). Finally, the immunization status of young children (which requires vaccine histories) and vaccine product information (e.g., inactivated compared with live attenuated) also were unavailable for this interim analysis. End-of-season VE estimates could change as additional patient data become available or if circulating viruses or population immunity change over the remainder of the season.

Although imperfect, influenza vaccines remain the best tool currently available for preventing illness from influenza. This report highlights the value of both increasing the use of influenza vaccines, especially among children and young adults, and continuing efforts to develop more effective vaccines and vaccination strategies. Antiviral medications are important for the treatment and control of influenza and should be used as recommended, regardless of patient vaccination status.

What is already known on this topic?

Annual vaccination is the mainstay of influenza prevention, but overall effectiveness of the influenza vaccine is moderate and varies by year, virus type, and population subgroup. Early unadjusted interim estimates of overall vaccine effectiveness (VE) for the 2012–13 season indicated the vaccine was 62% effective among all ages at preventing medically attended, laboratory-confirmed influenza A and B virus infections.

What is added by this report?

This report provides updated and adjusted VE estimates for the 2012–13 influenza season based on data from 2,697 children and adults with acute respiratory illness enrolled in the U.S. Influenza Vaccine Effectiveness (Flu VE) Network during December 3, 2012–January 19, 2013. The overall VE (adjusted for age group, study site, race/ethnicity, self-rated health status, and days from illness onset to enrollment) for all ages at preventing medically attended influenza A and B virus infections was 56% (95% confidence interval = 47%–63%). VE was estimated at 47% against influenza A (H3N2) virus infections and 67% against influenza B virus infections. VE against influenza A (H3N2) was lower and not statistically significant among adults aged ≥65 years.

What are the implications for public health practice?

The 2012–13 seasonal influenza vaccine provides substantial protection for the population overall, which underscores the public health value of vaccination. Nonetheless, some vaccinated persons have become ill with influenza this season, especially among persons aged ≥65 years. Antiviral medications are an important second line of defense against influenza and should be used promptly, as recommended for treatment of suspected influenza in certain patients in high-risk groups, including those aged ≥65 years, regardless of their vaccination status.

## Figures and Tables

**FIGURE:**
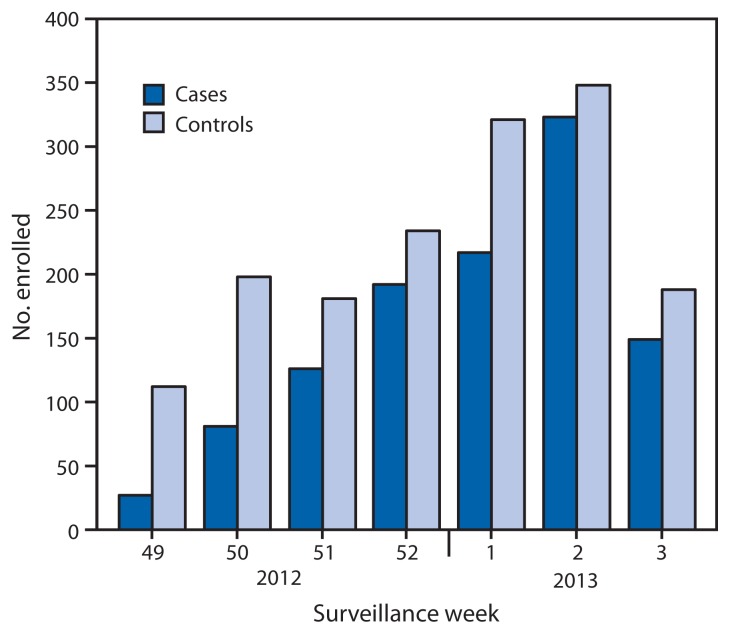
Numbers of influenza-positive cases and influenza-negative controls, by surveillance week of illness onset — U.S. Influenza Vaccine Effectiveness Network, United States, December 3, 2012–January 19, 2013 * Week 3 includes only patients with completed laboratory tests and thus does not reflect all enrolled patients during that week across study sites.

**TABLE 1:** Selected characteristics for enrolled patients with medically attended acute respiratory illness, by infuenza test result status and seasonal influenza vaccination status — U.S. Influenza Vaccine Effectiveness Network,* United States, December 3, 2012–January 19, 2013

	Test result status					Vaccination status		
		
	Influenza-negative		Influenza-positive			Vaccinated§		
					
Characteristic	No.	(%)	No.	(%)	p-value†	No./Total	(%)	p-value†
**Overall**	**1,582**	**(100)**	**1,115**	**(100)**		**1,160/2,697**	**(43)**	
**Study site**					**<0.001**			**<0.001**
Michigan	257	(16)	138	(12)		**168/395**	(43)	
Pennsylvania	360	(23)	208	(18)		**251/568**	(44)	
Texas	452	(29)	251	(23)		**254/703**	(36)	
Washington	173	(11)	90	(8)		**142/263**	(54)	
Wisconsin	340	(22)	428	(39)		**345/768**	(44)	
**Sex**					**0.358**			**0.006**
Male	629	(40)	463	(42)		**435/1,092**	(40)	
Female	953	(60)	652	(58)		**725/1,605**	(45)	
**Age group (yrs)**					**<0.001**			**<0.001**
6 mos–8	379	(24)	261	(23)		**275/640**	(43)	
9–17	186	(12)	202	(18)		**118/388**	(30)	
18–49	604	(38)	353	(32)		**356/957**	(37)	
50–64	248	(16)	174	(16)		**206/422**	(49)	
≥65	165	(10)	125	(11)		**205/290**	(71)	
**Race/Ethnicity** ¶					**0.006**			**0.012**
White	1,191	(75)	885	(80)		**922/2076**	(44)	
Hispanic	154	(10)	94	(8)		**88/248**	(36)	
Black	137	(9)	60	(5)		**72/197**	(37)	
Other race	100	(6)	76	(7)		**78/176**	(44)	
**Self-rated health status**					**<0.001**			**<0.001**
Fair or poor	138	(9)	68	(6)		**104/206**	(50)	
Good	405	(26)	236	(21)		**297/641**	(46)	
Very good	557	(35)	378	(34)		**424/935**	(45)	
Excellent	482	(30)	433	(39)		**335/915**	(37)	
**Illness onset to enrollment (days)**					**<0.001**			**0.061**
<3	544	(34)	504	(45)		**441/1,048**	(42)	
3–4	653	(41)	410	(37)		**442/1,063**	(42)	
5–7	385	(24)	201	(18)		**277/586**	(47)	
**Influenza test result**								
Negative	1,582	(100)	—	—		**793/1,582**	(50)	
Influenza B positive**	—	—	366	(33)		**90/366**	(25)	
Influenza A positive**	—	—	751	(67)		**277/751**	(37)	
A (H1N1)pdm	—	—	14	(2)		**2/14**	(14)	
A (H3N2)	—	—	546	(73)		**211/546**	(39)	
A subtype pending	—	—	191	(15)		**64/191**	(34)	

**Abbreviation:** rRT-PCR = real-time reverse transcription–polymerase chain reaction.

* The five network sites and the dates enrollment began were as follows: Group Health Cooperative (Seattle, Washington) (December 26, 2012); the Marshfield Clinic Research Foundation (Marshfield, Wisconsin) (December 17, 2012); the University of Michigan School of Public Health, partnered with the University of Michigan Health System (Ann Arbor, Michigan) (December 17, 2012) and the Henry Ford Health System (Detroit, Michigan) (January 2, 2013); the University of Pittsburgh Schools of the Health Sciences, partnered with the University of Pittsburgh Medical Center (Pittsburgh, Pennsylvania) (December 3, 2012); and Scott and White Healthcare (Temple, Texas) (December 9, 2012).

† Chi-square testing was used to assess differences between persons with influenza-negative and influenza-positive test results and in the distribution of enrolled patient and illness characteristics and also to assess differences between groups in the percentage vaccinated.

§ Defined as having received ≥1 dose of vaccine ≥14 days before illness onset. To date, 92% of influenza vaccines administered to participants have been inactivated. A total of 40 participants who received the vaccine ≤13 days before illness onset were excluded from the study sample because of uncertain immunization status.

¶ Enrollees were categorized into one of four mutually exclusive racial/ethnic populations: white, black, other race, and Hispanic. Persons identified as Hispanic might be of any race. Persons identified as white, black, or other race are non-Hispanic. The overall prevalences calculated included data from all racial/ethnic groups, not just the three included in this analysis.

** Two case-patients had coinfections with influenza A and B, making the sum 1,117, or two greater than the total number of influenza positives.

**TABLE 2:** Number and percentage receiving 2012–13 seasonal trivalent influenza vaccine among 2,697 outpatients with acute respiratory illness and cough, by influenza test result status, age group, and vaccine effectiveness* against all influenza A and B and against virus types A (H3N2) and B — U.S. Influenza Vaccine Effectiveness Network,† United States, December 3, 2012–January 19, 2013

					Vaccine effectiveness			
			
	Influenza-positive		Influenza-negative		Unadjusted		Adjusted	
				
Influenza type/Age group	No. vaccinated/Total	(%)	No. vaccinated/Total	(%)	(%)	(95% CI)	(%)	(95% CI)
**Influenza A and B**								
**Overall**	**367/1,115**	**(33)**	**793/1,582**	**(50)**	**(51)**	**(43–58)**	**(56)**	**(47–63)**
**Age group (yrs)**								
6 mos–17	118/463	(26)	275/565	(49)	(64)	(53–72)	(64)	(51–73)
18–49	100/353	(28)	256/604	(42)	(46)	(29–60)	(52)	(38–79)
50–64	63/174	(36)	143/248	(58)	(58)	(38–72)	(63)	(43–76)
≥65	86/125	(69)	119/165	(72)	(15)	(−42 to 49)	(27)	(−31 to 59)
**Influenza A (H3N2) only**								
**Overall**	**211/544**	**(39)**	**793/1,582**	**(50)**	**(37)**	**(23–48)**	**(47)**	**(35–58)**
**Age group (yrs)**								
6 mos–17	52/179	(29)	275/565	(49)	(57)	(38–70)	(58)	(38–71)
18–49	53/183	(29)	256/604	(42)	(45)	(21–61)	(46)	(20–63)
50–64	41/96	(43)	143/248	(58)	(45)	(12–66)	(50)	(15–71)
≥65	65/86	(76)	119/165	(72)	(−20)	(−118 to 34)	(9)	(−84 to 55)
**Influenza B only**								
**Overall**	**90/364**	**(25)**	**793/1,582**	**(47)**	**(67)**	**(58–77)**	**(67)**	**(51–78)**
**Age group (yrs)**								
6 mos–17	59/230	(26)	275/565	(49)	(64)	(49–74)	(64)	(46–75)
18–49	17/79	(22)	256/604	(42)	(63)	(35–79)	(68)	(40–83)
50–64	8/40	(20)	143/248	(58)	(82)	(59–92)	(75)	(39–90)
≥65	6/15	(40)	119/165	(72)	(74)	(24–91)	(67)	(−10 to 90)

**Abbreviation:** CI = confidence interval.

* Vaccine effectiveness was estimated as 100% × (1 – odds ratio [ratio of odds of being vaccinated among outpatients with influenza-positive test results to the odds of being vaccinated among outpatients with influenza-negative test results]); odds ratios were estimated using logistic regression.

† The five network sites and the dates enrollment began were as follows: Group Health Cooperative (Seattle, Washington) (December 26, 2012); the Marshfield Clinic Research Foundation (Marshfield, Wisconsin) (December 17, 2012); the University of Michigan School of Public Health, partnered with the University of Michigan Health System (Ann Arbor, Michigan) (December 17, 2012) and the Henry Ford Health System (Detroit, Michigan) (January 2, 2013); the University of Pittsburgh Schools of the Health Sciences, partnered with the University of Pittsburgh Medical Center (Pittsburgh, Pennsylvania) (December 3, 2012), and Scott and White Healthcare (Temple, Texas) (December 9, 2012).

## Footnotes

* The five network sites and the dates enrollment began were as follows: Group Health Cooperative (Seattle, Washington) (December 26, 2012); the Marshfield Clinic Research Foundation (Marshfield, Wisconsin) (December 17, 2012); the University of Michigan School of Public Health, partnered with the University of Michigan Health System (Ann Arbor, Michigan) (December 17, 2012) and the Henry Ford Health System (Detroit, Michigan) (January 2, 2013); the University of Pittsburgh Schools of the Health Sciences, partnered with the University of Pittsburgh Medical Center (Pittsburgh, Pennsylvania) (December 3, 2012); and Scott and White Healthcare (Temple, Texas) (December 9, 2012).

† A CDC influenza update for geriatricians and other clinicians caring for persons aged ≥65 years is available at http://www.cdc.gov/flu/professionals/2012-2013-guidance-geriatricians.htm.

§ Guidance for clinicians on antiviral use is available at http://www.cdc.gov/flu/professionals/antivirals/summary-clinicians.htm.
